# IL-1 Mediates Chronic Stress-Induced Hyperalgesia Accompanied by Microglia and Astroglia Morphological Changes in Pain-Related Brain Regions in Mice

**DOI:** 10.3390/ijms24065479

**Published:** 2023-03-13

**Authors:** Barbara Fülöp, Ágnes Hunyady, Noémi Bencze, Viktória Kormos, Nikolett Szentes, Ádám Dénes, Nikolett Lénárt, Éva Borbély, Zsuzsanna Helyes

**Affiliations:** 1 Department of Pharmacology and Pharmacotherapy, Medical School & Centre of Neuroscience, University of Pécs, H-7624 Pécs, Hungary; 2 GSK Vaccines Institute for Global Health, I-53100 Siena, Italy; 3 “Momentum” Laboratory of Neuroimmunology, Institute of Experimental Medicine, H-1083 Budapest, Hungary; 4 Eotvos Lorand Research Network, Chronic Pain Research Group, University of Pécs, H-7624 Pécs, Hungary; 5 National Laboratory for Drug Research and Development, H-1117 Budapest, Hungary

**Keywords:** cytokines, neuroinflammation, hyperalgesia, chronic immobilization, fibromyalgia-like pain syndrome, central pain sensitization

## Abstract

Chronic stress causes several pain conditions including fibromyalgia. Its pathophysiological mechanisms are unknown, and the therapy is unresolved. Since the involvement of interleukin-1 (IL-1) has been described in stress and inflammatory pain but no data are available regarding stress-induced pain, we studied its role in a chronic restraint stress (CRS) mouse model. Female and male C57Bl/6J wild-type (WT) and IL-1αβ-deficient (knock-out: IL-1 KO) mice were exposed to 6 h of immobilization/day for 4 weeks. Mechanonociception, cold tolerance, behavioral alterations, relative thymus/adrenal gland weights, microglia ionized calcium-binding adaptor molecule 1 (IBA1) and astrocyte glial fibrillary acidic protein (GFAP) integrated density, number and morphological transformation in pain-related brain regions were determined. CRS induced 15–20% mechanical hyperalgesia after 2 weeks in WT mice in both sexes, which was significantly reduced in female but not in male IL-1 KOs. Increased IBA1+ integrated density in the central nucleus of amygdala, primary somatosensory cortex hind limb representation part, hippocampus cornu ammonis area 3 (CA3) and periaqueductal gray matter (PAG) was present, accompanied by a cell number increase in IBA1+ microglia in stressed female WTs but not in IL-1 KOs. CRS induced morphological changes of GFAP+ astrocytes in WT but not in KO mice. Stress evoked cold hypersensitivity in the stressed animals. Anxiety and depression-like behaviors, thymus and adrenal gland weight changes were detectable in all groups after 2 but not 4 weeks of CRS due to adaptation. Thus, IL-1 mediates chronic stress-induced hyperalgesia in female mice, without other major behavioral alterations, suggesting the analgesic potentials of IL-1 in blocking drugs in stress-related pain syndromes.

## 1. Introduction

Chronic primary pain is a subtype of chronic pain characterized by pain of unknown origin in one or more anatomic regions that persists or recurs for longer than 3 months [1]. The term is used in the International Classification of Diseases (ICD) guideline published by the World Health Organization (WHO) [2,3]. It is associated with significant emotional distress (anxiety and depression) and often leads to functional disability in daily life, also interfering with participation in social roles [4]. This kind of pain cannot be fully explained by any other chronic pain condition. The most common chronic primary pain diseases are complex regional pain syndrome (CRPS) and fibromyalgia [3,5]. Although trauma in CRPS can precede the development of the disease, the surfacing pain is disproportional to the underlying organic lesion [1,2]. These diseases still have no specific therapy, and despite complex treatment (CRPS and fibromyalgia are treated with antidepressants and anticonvulsants, as well as physiotherapy [6,7]), they still lead to a great reduction in the quality of life.

Fibromyalgia is a chronic primary pain syndrome of non-inflammatory origin, mainly affecting the muscles and soft tissues [8]. This disease, which occurs in 3–6% of the population, affects women in 70–90% [5,9]. Among the symptoms, there are fatigue, depression, memory loss, widespread pain, and sleeping disturbances. The disease is characterized by a complex intergrowth of biological, psychological and social factors [1]. This is the reason why translation to an animal model is difficult. Although the pathogenesis of the disease is an area of intense research, the exact molecular mechanisms by which the disease develops are still unknown. As far as fibromyalgia’s etiology is undiscovered, the disease does not have a well-established animal model that mimics all the signs and symptoms of the complex illness. Patients with fibromyalgia have, in addition to their spontaneous widespread pain, disproportionally increased pain in response to painful stimuli (“mechanical hyperalgesia”) [10,11,12]. 

The reduction in the mechanical pain threshold due to chronic immobilization stress has already been described in rodent models [13,14]. Therefore, a potential approach to investigating fibromyalgia-like pain sensitization is to examine the stress-induced nociceptive changes in a chronic stress model applied to both female and male mice. Beyond stress factors, the involvement of local neuroinflammatory mechanisms might further complicate the pathophysiology of the disease. 

Neuroinflammation is the innate immunological response to injury (disease, trauma, stress and toxins) in the central nervous system, characterized by altered microglia and astrocyte activity [15]. Microglia may shift their activity states in response to noxious stimuli depending on their strength and nature, which may lead to increased cell numbers, morphological transformation and an increased release of inflammatory mediators [16,17,18]. Similar to microglia, astrocytes are also involved in neuroinflammatory mechanisms via complex interactions [19,20]. The activation of these cells plays a crucial role in fibromyalgia [21,22]. 

Interleukine-1 (IL-1) was discovered in the 1940s [23] in relation to fever, but the current name was given in 1979 based on its divergent functions in inflammatory cascades [24]. The first-described isoforms, IL-1 α and β, are still the most thoroughly investigated pro-inflammatory cytokines of this 11-member family. IL-1 in the CNS is produced predominantly by macrophages and microglia but also by endothelial and epithelial cells, acting on several targets, including astrocytes [19,25,26].

The increase in the circulating amount of IL-1β following acute psychological stress demonstrates its role in stress responses [27]. Clinical studies have suggested that IL-1 contributes to inflammation in rheumatoid arthritis and osteoarthritis [28,29]. Furthermore, IL-1 has already been associated with the development of depression [30].

Until now, IL-1 has been shown to be involved in mood disorders and has been linked with stress and inflammatory pain. However, there are still no data available regarding their role in stress-induced pain syndromes. Therefore, in our study, we aimed to find out the possible involvement of IL-1 in a female and male mouse model of chronic restraint stress-induced pain.

## 2. Results

### 2.1. Female Mice Are More Sensitive to Mechanical Stimuli but Less Sensitive to Cold Stimuli than Males

**Figure 1:**
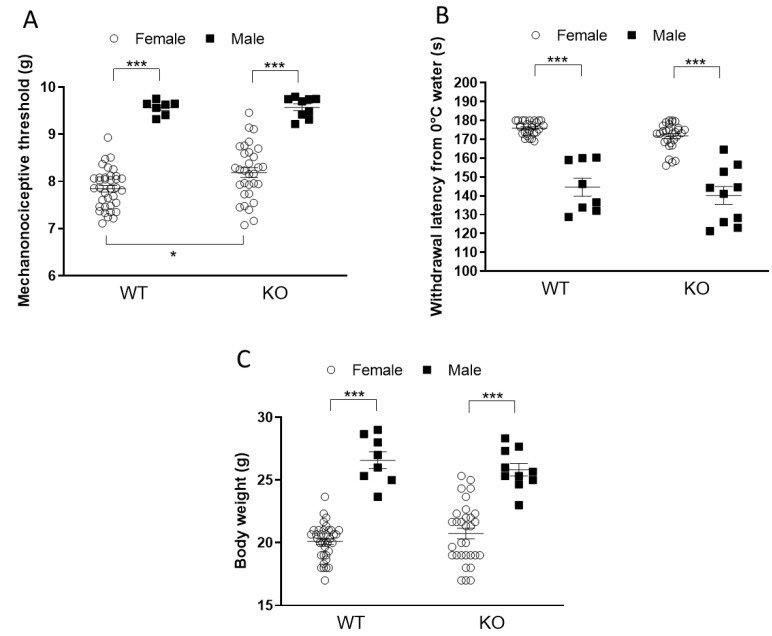
Baseline mechanonociceptive threshold (**A**), cold tolerance (**B**) and body weight (**C**) values of female and male wild-type (WT) and Interleukine-1-deficient (knock-out: KO) mice at the beginning of the experiment. Data are presented as the means ± SEM of *n* = 7–36 animals with individual plots (female WT: 36; female KO: 31; male WT: 7; male KO: 9); two-way analysis of variance (ANOVA), followed by Tukey’s tests; * *p* < 0.05, *** *p* < 0.0001 vs. indicated groups.

Significant differences were detected between the two sexes in the baseline nociceptive measurements. Lower mechanonociceptive thresholds (Figure 1A)—but, interestingly, a higher cold tolerance (Figure 1B)—were measured in females compared to males in both WT and IL-1 KO mice. In the case of the females, IL-1 deficiency resulted in significantly higher mechanonociceptive thresholds but did not influence the cold sensitivity (Figure 1A,B). The body weights of the female mice of both genotypes at the age of 12–14 weeks were significantly lower compared to those of males (Figure 1C).

### 2.2. Chronic Restraint Stress (CRS)-Induced Mechanical Hyperalgesia, but Not Cold Hyperalgesia, Is Significantly Reduced in Female IL-1 KO Mice 

**Figure 2:**
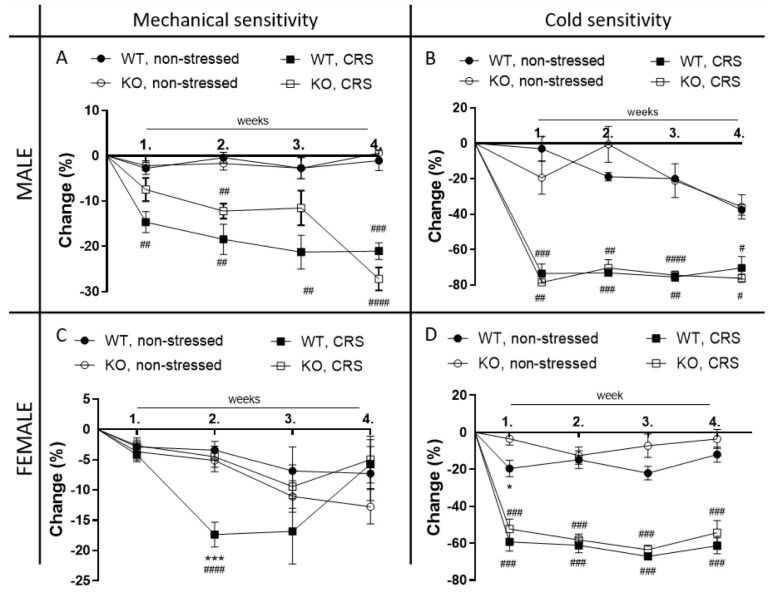
Effects of chronic restraint stress (CRS) on nociceptive behaviors of wild-type (WT) and Interleukine-1 knock-out (KO) mice. Male (**A**) and female (**C**) mechanonociceptive threshold and male (**B**) and female (**D**) cold tolerance (paw withdrawal latency) changes are presented compared to the baseline threshold. Data are presented as the means ± SEM (*n* = 4–19); two-way repeated measurement analysis of variance (ANOVA), followed by Tukey’s tests; *** *p* < 0.001 vs. KO stressed group; * *p* < 0.05 vs. KO non-stressed group; # *p* < 0.05, ## *p* < 0.01, ### *p* < 0.001, #### *p* < 0.0001 vs. respective non-stressed groups.

Both male and female mice developed an approximately 15–20% decrease in the mechanonociceptive threshold (mechanical hyperalgesia) and a 60–70% cold sensitivity increase in response to CRS from the first week. Mechanical hyperalgesia was maintained during the 4 weeks of the experiment in males but normalized after 3 weeks in females (Figure 2A,C). Cold hypersensitivity remained unchanged during the whole study in all groups (Figure 2B,D). IL-1 deficiency did not influence stress-induced mechanical and cold hyperalgesia in male mice (Figure 2B) but significantly reduced mechanical hyperalgesia in females at 2 weeks without influencing their cold hypersensitivity (Figure 2C,D). 

### 2.3. CRS-Induced Relative Thymus and Adrenal Weight Changes at 2 Weeks Vanished after 4 Weeks of CRS

**Figure 3:**
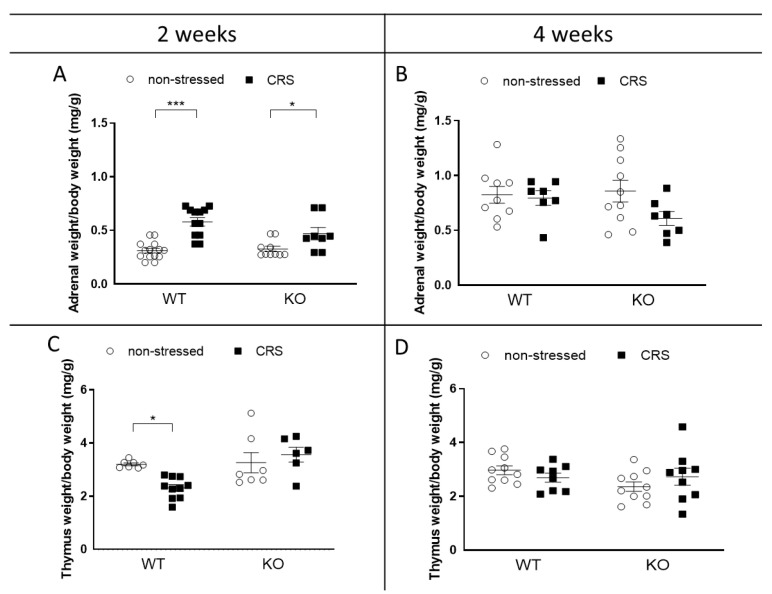
Chronic restraint stress (CRS)-induced weight changes of stress-sensitive organs in wild-type (WT) and Interleukine-1 knock-out (KO) female animals. Thymus weights after 2 weeks (**A**) and 4 weeks (**B**); adrenal gland weights after 2 weeks (**C**) and 4 weeks (**D**) of CRS. Data are presented as the means ± SEM of *n* = 6–12 animals with individual plots; two-way analysis of variance (ANOVA), followed by Tukey’s tests; * *p* < 0.05, *** *p* < 0.001 vs. respective control group.

Female animals’ relative adrenal weights (total adrenal weight (mg)/body weight (g)) were increased in both genotypes due to 2 weeks of CRS (Figure 3A), and the relative thymus weights (thymus weight (mg)/body weight (g)) were decreased in the WTs but not in the KO groups (Figure 3C). Every difference vanished by the 4th week of stress (Figure 3B,D). 

### 2.4. CRS-Induced Immobility Decreases in KO but Not in WT Mice after 2 Weeks of CRS

#### 2.4.1. Depression-like Behavior Changes following CRS in Female Animals

**Figure 4:**
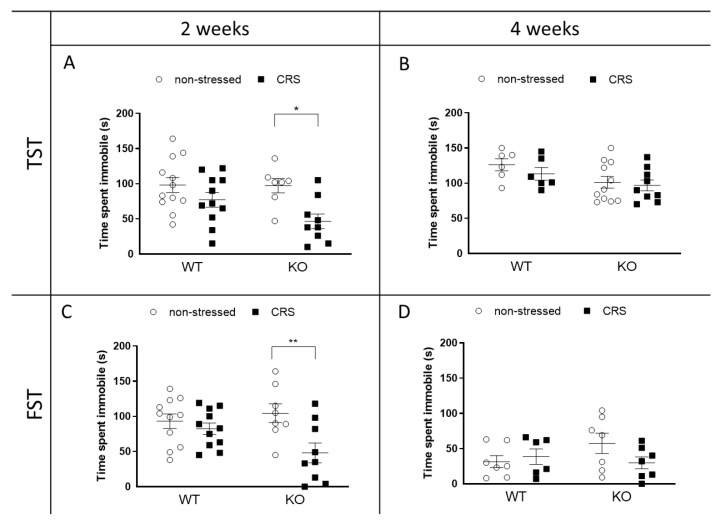
Effects of chronic restraint stress (CRS) on depression-like behavior in wild-type (WT) and Interleukine-1 knock-out (KO) female animals. Tail suspension test (TST) shows time spent immobile after 2 weeks (**A**) and 4 weeks (**B**) of restraint. Immobility time shown in the forced swim test (FST) after 2 weeks (**C**) and after 4 weeks (**D**) of CRS. Data are presented as the means ± SEM of *n* = 6–12 animals with individual plots; two-way analysis of variance (ANOVA), followed by Tukey’s tests; * *p* < 0.05, ** *p* < 0.01 vs. own control group.

After 2 weeks, a significant immobility time drop was detectable in the IL-1 KO, due to stress in both the Forced swim test (FST) and Tail suspension test (TST) (Figure 4A,C). This drop did not reach the level of significance in the WT groups. After 4 weeks, this difference between the four groups vanished in both tests (Figure 4B,D).

#### 2.4.2. CRS Induced No Alteration in Anxiety-like Behaviors in Female Mice

##### Light–Dark Box (LDB) Test

**Figure 5:**
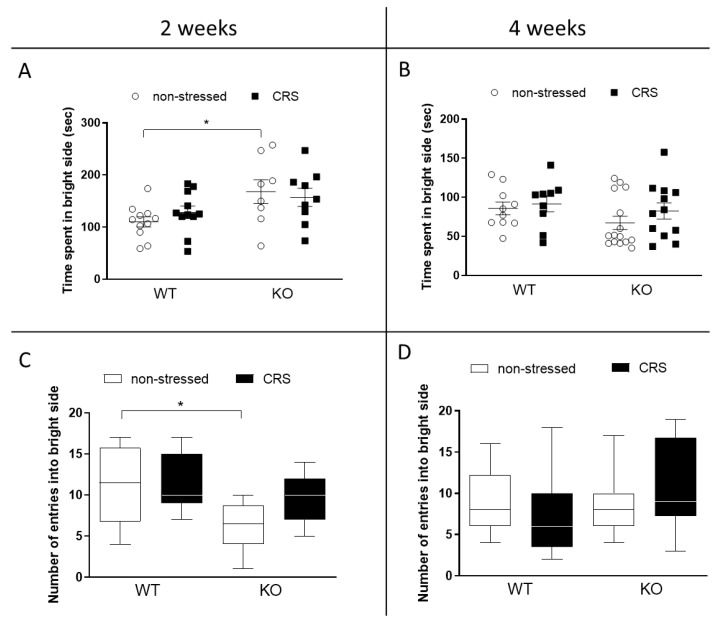
Behavioral alterations induced by chronic restraint stress (CRS) in female wild-type (WT) and Interleukine-1 knock-out (KO) mice detected with the light–dark box test (LDB). Time spent in the lit compartment after 2 weeks (**A**) and after 4 weeks (**B**); the number of entries into the light after 2 weeks (**C**) and 4 weeks (**D**). Data are presented as the means ± SEM in case of A&B (animals with individual plots) and as the median ± IQR in case of C&D (*n* = 8–15); two-way analysis of variance (ANOVA), followed by Tukey’s tests; Kruskal–Wallis test, followed by Dunn’s test; * *p* < 0.05 vs. indicated group.

Neither the time spent in the light compartment, nor the number of entries were affected by the CRS after 2 weeks (Figure 5A,C) or 4 weeks (Figure 5B,D). There was an indicated difference between the non-stressed groups at 2 weeks; the IL-1 KO group spent significantly more time and entered the bright side less compared to the WT ones.

##### Open Field Test (OFT) 

**Figure 6:**
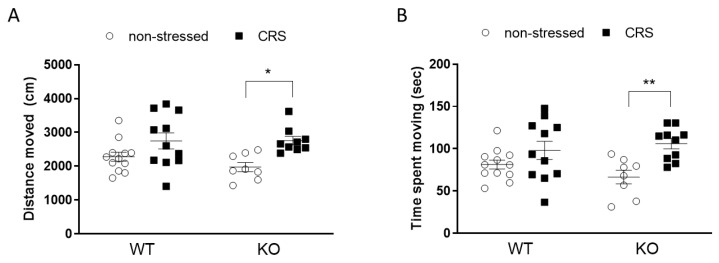
Behavioral alterations induced by chronic restraint stress (CRS) in wild-type (WT) and Interleukine-1 knock-out (KO) female mice detected with an open field test (OFT). The distance moved (**A**) and the time spent on moving (**B**) in the OFT. Data are presented as the means ± SEM of *n* = 8–12 animals with individual plots; two-way analysis of variance (ANOVA), followed by Tukey’s tests; * *p* < 0.05, ** *p* < 0.01 vs. own control group.

Two weeks of stress induced a significant locomotor activity increase in the IL-1 KO animals but not in the WTs (Figure 6A,B). 

### 2.5. CRS-Induced Microglia and Astrocyte Changes in Pain-Related Brain Regions of WT and IL-1 KO Female Mice

#### 2.5.1. CRS-Induced Higher Integrated Density of Ionized Calcium-Binding Adaptor Molecule 1 (IBA1)-Positive Microglia in Pain-Related Brain Areas with a Greater IBA1+ Microglia Cell Count in the Periaqueductal Gray (PAG)

**Figure 7:**
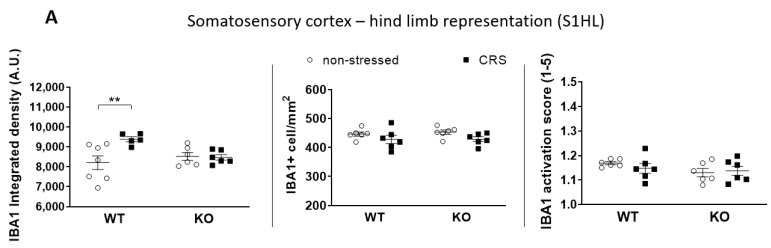

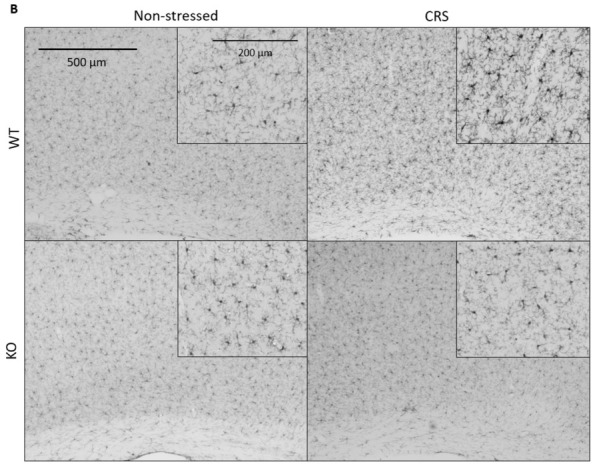
Diagrams show the ionized calcium-binding adaptor molecule 1 (IBA1) integrated density, the number of IBA1+ cells and the IBA1 activation score of microglia examined in the somatosensory cortex–hind limb representation part (S1HL—(**A**)). Representative images show the IBA1+ microglia cells in S1HL (**B**). Chronic restraint stress (CRS), Wild-type (WT), Interleukine-1 knock-out (KO). Data are presented as the means ± SEM of *n* = 5–7 animals with individual plots; two-way analysis of variance (ANOVA), followed by Tukey’s tests; ** *p* < 0.01 vs. respective group.

**Figure 8:**
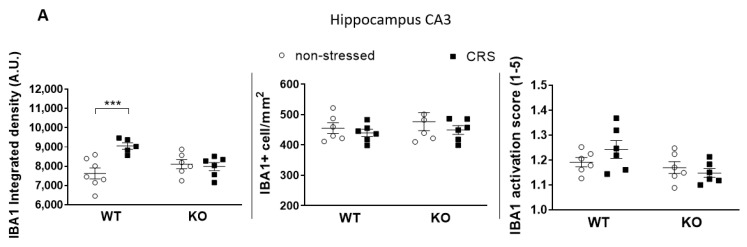

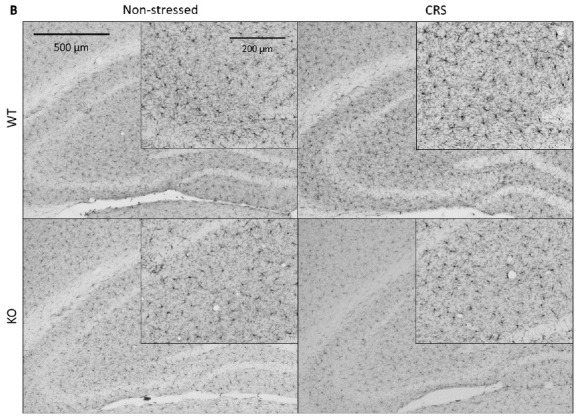
Diagrams show the Ionized calcium-binding adaptor molecule 1 (IBA1) integrated density, the number of IBA1+ cells and the IBA1 activation score of microglia examined in the hippocampus CA3 region (CA3—(**A**)). Representative images show the IBA1+ microglia cells in CA3 (**B**). Chronic restraint stress (CRS), Wild-type (WT), Interleukine-1 knock-out (KO). Data are presented as the means ± SEM of *n* = 5–7 animals with individual plots; two-way analysis of variance (ANOVA), followed by Tukey’s tests; *** *p* < 0.001 vs. respective group.

**Figure 9:**
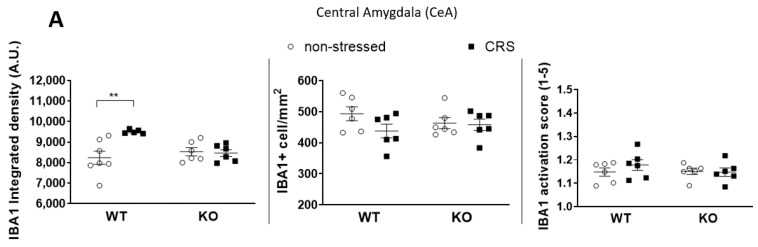

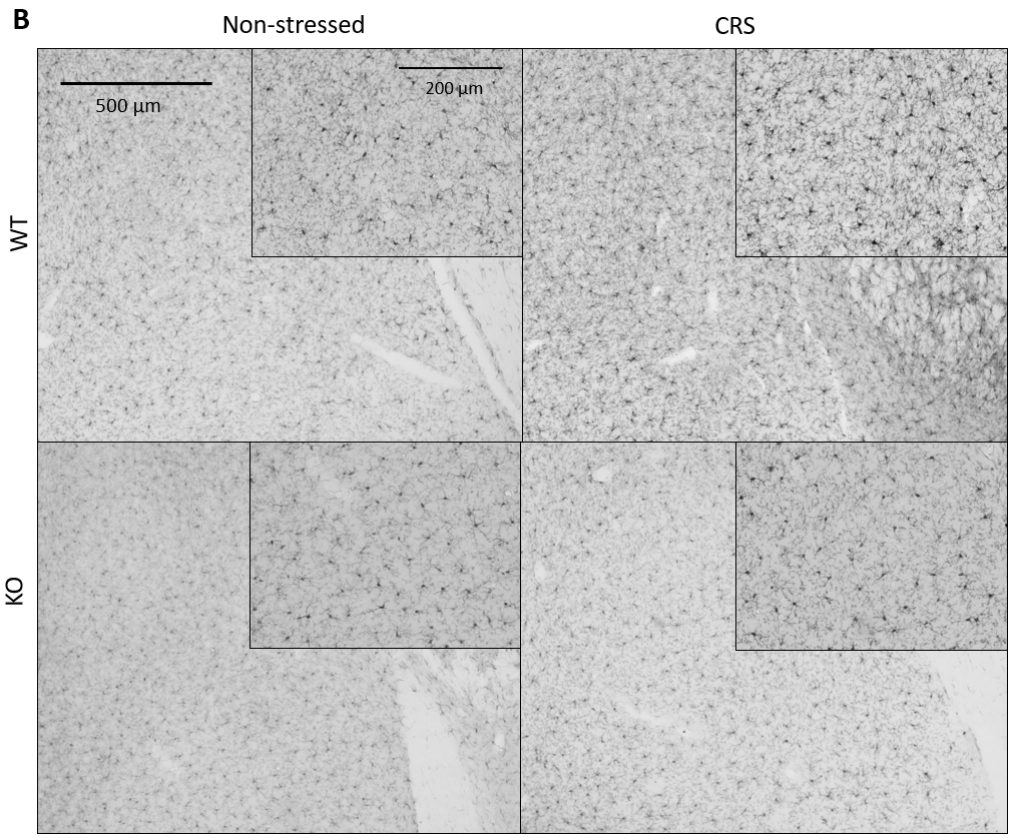
Diagrams show the Ionized calcium-binding adaptor molecule 1 (IBA1) integrated density, the number of IBA1+ cells and the IBA1 activation score of microglia examined in the Central Amygdala (CeA—(**A**)). Representative images show the IBA1+ microglia cells in CeA (**B**). Chronic restraint stress (CRS), Wild-type (WT), Interleukine-1 knock-out (KO). Data are presented as the means ± SEM of *n* = 5–7 animals with individual plots; two-way analysis of variance (ANOVA), followed by Tukey’s tests; ** *p* < 0.01 vs. respective group.

**Figure 10:**
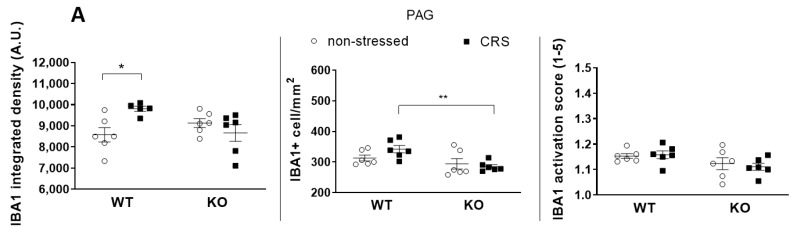

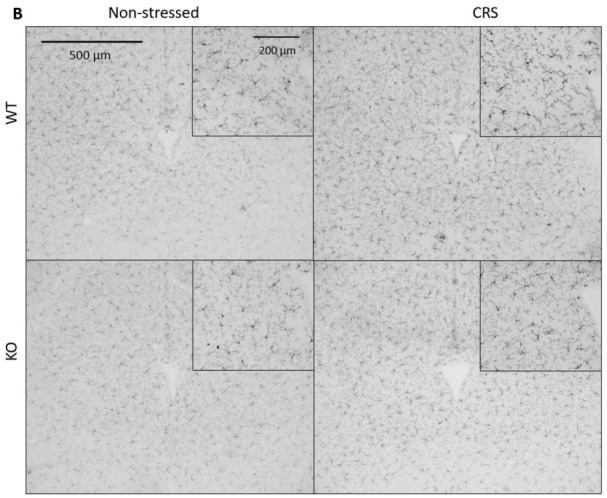
Diagrams show the Ionized calcium-binding adaptor molecule 1 (IBA1) integrated density, the number of IBA1+ cells and the IBA1 activation score of microglia examined in the Periaqueductal gray (PAG—(**A**)). Representative images show the IBA1+ microglia cells in PAG (**B**). Chronic restraint stress (CRS), Wild-type (WT), Interleukine-1 knock-out (KO). Data are presented as the means ± SEM of *n* = 5–7 animals with individual plots; two-way analysis of variance (ANOVA), followed by Tukey’s tests; * *p* < 0.05, ** *p* < 0.01 vs. respective group.

The baseline integrated IBA1 densities were similar in all investigated brain areas in both groups (Figure 7A, Figure 8A, Figure 9A and Figure 10A). After 2 weeks of CRS, a significantly higher integrated density was detected in stressed WT but not in KO mice (Figure 7B, Figure 8B, Figure 9B and Figure 10B).

The IBA1+ cell number in the PAG was significantly higher after 2 weeks of CRS in WT mice compared to that in the KO ones. However, there was no difference between the activation scores of the four groups (Figure 10A). No differences were detected in IBA1+ cell numbers or microglia activation scores after 2 weeks of CRS between any groups in any other investigated brain regions (Figure 7A, Figure 8A and Figure 9A).

#### 2.5.2. CRS-Induced Glial Fibrillary Acidic Protein (GFAP)-Positive Astroglia Activation in the Hippocampal CA3

**Figure 11:**
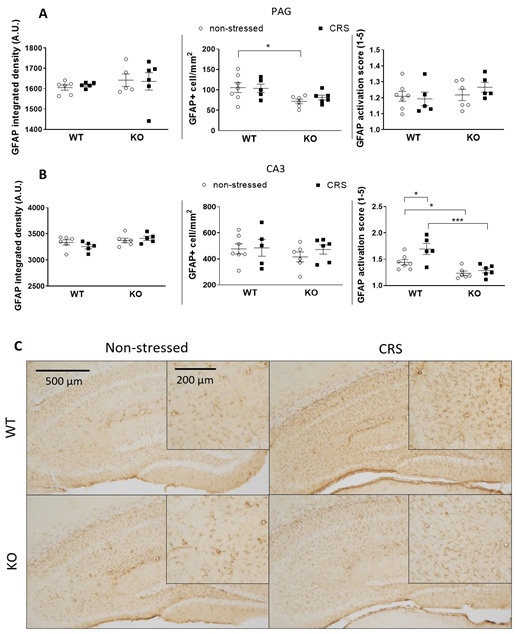
Diagrams show the Glial fibrillary acidic protein (GFAP) integrated density, the number of GFAP+ cells and the GFAP activation score of astrocytes examined in the periaqueductal gray matter (PAG—(**A**)) and the hippocampus cornu ammonis area 3 (CA3—(**B**)). Representative images show the GFAP+ astroglia cells in CA3 (**C**). Chronic restraint stress (CRS), Wild-type (WT), Interleukine-1 knock-out (KO). Data are presented as the means ± SEM of *n* = 5–7 animals with individual plots; two-way analysis of variance (ANOVA), followed by Tukey’s tests; * *p* < 0.05, *** *p* < 0.001 vs. respective group.

No GFAP+ astrocytes were visible in the CeA and S1HL regions. Baseline GFAP+ cell numbers were significantly higher in the PAG of non-stressed WT animals compared to those in the IL-1 KOs. CRS had no significant effect on the GFAP integrated density and activation scores (Figure 11A) in the PAG. In the hippocampal CA3, no difference was observed in the GFAP+ integrated density and in the cell numbers between the groups; however, CRS evoked significant cell activation in WT but not in IL-1 KO mice (Figure 11B,C). 

## 3. Discussion

Here, we provide the first evidence that IL-1 contributes to inflammatory changes in the brain, mediating chronic stress-induced pain. These changes may be indicated by altered microglia and astrocyte morphologies in stress- and pain-related brain regions of female mice. Importantly, remarkable sex differences in stress-induced pain- and mood-associated behavioral parameters were demonstrated.

In agreement with the literature data, in baseline measurements, female mice showed a lower sensitivity to cold and a greater susceptibility to mechanical stimuli, accompanied by a smaller body weight, compared to males [31,32,33]. No significant difference between the two genotypes was present in these parameters, except for mechanonociception. Female IL-1 KO mice had significantly higher pain thresholds compared to WTs. Several data suggest that IL-1 knockdown or IL-1 system/receptor malfunction is associated with reduced nociception [34,35,36,37]. It has been shown that IL-1β not only induces inflammation and increases the synthesis of several mediators sensitizing the nociceptors but also directly activates nociceptive C fibers. IL-1-induced action potential generation was described in rat skin/nerve preparation, suggesting the ability of IL-1 to stimulate sensory nerves and evoke consequent pain [38]. Type 1 IL-1 receptors are highly expressed in capsaicin-sensitive primary sensory neurons in the mouse dorsal root ganglia, also supporting the potential of IL-1 to directly activate the nociceptive pathway [39,40]. Furthermore, the selective deletion of IL-1R1 in capsaicin-sensitive nociceptors prevented the development of mechanical allodynia in chronic inflammatory pain mouse models [41].

IL-1 receptor 1 (IL-1R1) activation on neurons, glia and vascular-endothelial cells causes the phosphorylation of cellular proteins such as heat shock proteins and various kinases including protein kinase C (PKC) and mitogen-activated protein kinase (MAP kinase) [42,43]. The IL-1-activated transcription factor NFκB binds to the promoter regions of some proinflammatory cytokines, such as IL-6 and IL-8 [44]. The role of IL-1 in inflammation after ischemic brain injury and cerebrovascular inflammation in response to atherogenic diet and systemic vascular disease in mice has been demonstrated by Dénes and colleagues [45,46]. 

Regarding mood and stress regulation, increased IL-1 expressions were observed in the rat hippocampus in response to social isolation [47] and in the hypothalamus after immobilization [48]. The intraperitoneal administration of IL-1β induced anxiety-like behavior in mice [49]. Increased IL-1β levels in the cerebrospinal fluid of bipolar patients [50] and in the crevicular fluid samples of students after exam stress also suggest the roles of IL-1 in stress responses [51]. 

We described here that stress-induced mechanical hyperalgesia was significantly smaller in female but not in male IL-1 KO mice compared to the respective WTs. Besides the role of IL-1 in stress, several data indicate its involvement in pain processing. Intraplantar, intrathecal and intravenous IL-1 injections were able to induce mechanical hyperalgesia by exciting nociceptive fibers and cause anxiety- and depression-related behavioral alterations in rats [20,52,53]. Spared sciatic nerve injury-induced pain, memory deficits and depression-like behaviors were prevented by the peri-sciatic administration of IL-1β neutralizing antibodies in rats [53]. The baseline IL-1 concentration in the blood samples of patients with non-specific chronic low back pain was higher compared to that in asymptomatic controls, which positively correlated with the pain severity scores [54]. Sural nerve biopsy samples of neuropathic pain patients demonstrated increased IL-1 immunoreactivity positively correlating with the pain intensity [55]. A recent meta-analysis focusing on the concentration of IL-1 in fibromyalgia patients could not show any significant difference between the patients with fibromyalgia and the healthy controls, despite some individual studies with opposing trends [56]. The described sex difference in the mechanical hyperalgesia supports the human data demonstrating the higher prevalence and severity of several common chronic pain conditions with etiological stress factors (e.g., fibromyalgia, irritable bowel syndrome) in women [57,58]. Although the stress-induced pain model does not entirely mimic the human disease, our results are likely to have translational value in the investigation of female-predominant nociceptive mechanisms related to psychosocial stressors. Cortical differences have been described during the pain processing of female and male patients [59,60]. Although the estrous cycle has long been suggested to play a significant role in influencing pain [61,62], there is increasing evidence that the rapid hormonal alterations within the cycle do not substantially affect pain sensitivity [63,64]. Therefore, using female mice besides males has strongly been recommended recently in pain research for determining sex-dependent molecular differences [65,66]. 

Several data suggest links between sex hormones and cytokine production. Estrogen-enhanced IL-1β promoter activity was demonstrated in an hER + IL-1β-CAT+ macrophage cell line [67]. Foot shock stress increased IL-1 levels in the hypothalamus of female rats [68]. The loss of estrogen in postmenopausal women results in elevated cytokine levels, including IL-1, which is involved in osteoporosis [69]. 

Although CRS did not induce mechanical hyperalgesia in female IL-1 KO mice in our study, it resulted in similarly and significantly decreased cold tolerance in both genotypes and sexes. These findings suggest a role for IL-1 in stress-induced mechanical but not cold hypersensitivity. Mechanical hyperalgesia was shown to involve central sensitization mechanisms besides peripheral processes; cold hypersensitivity is caused mainly by peripheral sensitization [70]. Based on these results, we hypothesize the role of IL-1 in the development of central sensitization, which is supported by the genotype difference in the mechanical pain threshold. Furthermore, the lack of a genotype difference in cold tolerance suggests that IL-1 is not likely to be involved in the development of peripheral sensitization.

Damage to the central nervous system (infection, trauma, ischemia, stress, etc.) results in a rapid neuroinflammatory response, regulated by glial cells [71]. 

CRS induced significantly higher IBA1+ integrated density in all four investigated pain-related brain areas of WT mice, which was not present in the KO ones. However, neither the microglia cell count nor activation were significantly increased in response to CRS, but the stress-induced microglia number elevation in the PAG was significantly lower in IL-1 KO mice. PAG plays a crucial role in the integration of the ascending and descending inhibitory pain pathways [72]; therefore, the reduced responsiveness of microglia in this region in the case of IL-1 deficiency might have functional relevance. In rats, chronic restraint stress (14 days, 1 h/day) induced a significantly elevated microglia number in the PAG, cerebral cortex and hippocampus CA3 [16], and acute restraint stress combined with water immersion evoked microglia activation in the hypothalamus and hippocampus [17]. Microglia reactions after stressful interventions in the hippocampus are supported by dynamic alterations of both the number and activation of these cells in the chronic unpredictable stress paradigm with an initial (2–3 days) increase and subsequent (5 weeks) reduction [18]. Density measurements demonstrated significant differences between the stressed and non-stressed groups, while the quantification of the cell numbers and activation states were not sensitive enough to demonstrate subtle changes. The applied integrated density approach used provides unbiased means to correlate changes in avidin–biotin microglial activity markers with morphological- and inflammatory readouts [73]. Our results demonstrate that IL-1 might have a role in CRS-related microglia changes, which may contribute to central pain sensitization processes in female mice.

There are strong and complex interactions between microglia and astrocytes in neuroinflammatory mechanisms in which proinflammatory cytokines released from activated microglia cells are involved [74].

The GFAP+ astrocyte number was significantly lower in the PAG of IL-1 KO mice compared to that of WTs, independently of stress, suggesting their IL-1-dependent homeostatic function. Two weeks of stress induced significant astrocyte activation in the hippocampus CA3 of WT but not KO mice. Mild to moderate astrogliosis in response to any stressors such as trauma, innate immune activation or infections [75] is related to proliferation and hypertrophy [20,76,77]. The literature data regarding stress-related astrogliosis are inconsistent. Five weeks of psychosocial stress decreased the number of GFAP+ cells in the hippocampus in male tree shrews [78], while 6 days of activity stress increased these cells in male rats [79]. Immobilization for 4 days in male mice increased the hippocampal astrocyte number [80], but this intervention for 10 days in male rats did not alter this parameter [81]. Restraint stress decreased GFAP protein expression in the hippocampus after 21 days in female mice [82] but increased this after 10 days in males [83]. These contradictions might be due to the differences in species, gender, stressors and durations. In our model, no change in the integrated density was detectable. Still, significant activation-score elevation was present in the CA3 region, suggesting a potential role of IL-1 in stress-induced hippocampal astrocyte activation in female mice, which might be involved in central pain sensitization. 

The stress-induced changes in the thymus and adrenal gland weights seen after 2 weeks in our study, disappearing by the fourth week, are in line with the literature data, which is a possible consequence of habituation. 

Peripheral endocrine changes have long been described in response to stress, reflected by increased relative adrenal gland weights and decreased thymus weights [84]. Our results confirm these phenomena after 2 but not 4 weeks of CRS, which is likely to be due to adaptation mechanisms. One week of daily 1.5-hour restraint stress induced a significant thymus weight decrease in mice [85], and chronic variable stress (including restraint stress) for 2 weeks in rats induced a significantly increased adrenal weight and decreased thymus weight [86]. Chronic, daily 6-hour restraint stress has been described to cause habituation by day 21 in the corticosterone response, and this long-term CRS caused neither adrenal hypertrophy nor thymus atrophy in rats [87]. Meanwhile, 10 days of 1-hour daily restraint stress did not induce an adrenal or thymus weight alteration in a rat study [86]. 

FST and TST are routinely used to assess acute depression-like behaviors and to determine the effects of anxiolytics and antidepressants [88,89]. The OFT and the LDB are appropriate for investigating spontaneous locomotor activity and anxiety, respectively, in rodents [90]. Despite significant hyperalgesia development after 2 weeks of CRS, anxiety- and depression-like behaviors were not detected in WT mice. Interestingly, although stress did not induce hyperalgesia in IL-1 KO mice, these animals showed increased spontaneous locomotor activity, which was also reflected by decreased immobility in the FST and TST. These results suggest that IL-1 might decrease the susceptibility to stress-induced anxiety and locomotion, in contrast to its pain-sensitizing role. 

While we did not experience habituation in cases of cold tolerance, the results of the behavioral studies indicate that the pain and behavior parameters are different in terms of the formation time and the degree of habituation. These data also draw attention to the importance of the complex regulation between pain and mood disorders. All behavioral and endocrine alterations detected after 2 weeks of CRS in WT mice were absent after 4 weeks, demonstrating adaptation [13,91,92]. 

The lack of IL-1 α and β in the whole body of our KO mice from the embryonic age might compensatorily influence the expression of other inflammatory mediators, which is a general limitation of using total KO mice. 

It is concluded that IL-1 mediates chronic stress-induced pain in female mice, in which neuroinflammation, and consequent central sensitization, might be involved. Inhibiting the IL-1 receptor 1 (IL-1R1) might be a potential therapeutic opportunity in chronic primary pain conditions such as fibromyalgia and complex regional pain syndrome, which are more common in female patients. 

## 4. Materials and Methods

### 4.1. Animals

Experiments were performed on female and male IL-1 αβ gene-deficient KO mice backcrossed for 7–8 generations to C57Bl/6 mice. C57Bl/6 mice were used as wild-type (WT) controls, and the original breeding pairs were purchased from Charles River Ltd.(Wilmington, MA, USA) IL-1 αβ KO mice were generated at the University of Budapest, Institute of Experimental Medicine, as previously described [93]. We have not observed any remarkable differences in global IL-1α/β KO mice as compared to their WTs regarding their development, growth, general health, lifespan and fertility, which is well supported by the literature data [94,95]. These mice can be bred and maintained in routine animal facilities; they do not exhibit evidence of immunosuppression and spontaneous carcinogenesis [95]. The animals were bred and kept in the Laboratory Animal House of the Department of Pharmacology and Pharmacotherapy Institute at the University of Pécs at 24–25 °C and 65% humidity, provided with standard mouse chow and water ad libitum and maintained under a 12 h light–dark cycle, starting at 7:00 a.m. The mice were housed in groups of two to five in polycarbonate cages (330 cm^2^ floor space, 12 cm height) on wood shaving bedding. Behavioral tests and perfusion were carried out in the laboratory of the Department at least 2 h after the last stress. 

### 4.2. The CRS Paradigm

The mice were restrained for 6 h every day by placing them into 50 mL well-ventilated plastic tubes with holes, in which their movements were restricted. Restraint stress routinely began in the morning. Non-stressed mice were identically handled, but they were not restrained and were left in their home cages in the Laboratory Animal House [96]. 

### 4.3. Experimental Design

Baseline nociceptive measurements were started 1 week before the CRS period. All initial nociceptive tests were performed three times on two non-consecutive days. Thereafter, the results were averaged and used as ‘Baseline’ values. During the 4-week-long CRS, nociceptive tests were performed once a week, whereas behavioral tests were implemented only once, on the week of termination. Male animals went through 4 weeks of CRS; one part of the female animals was terminated after 2 weeks, and the other part was terminated after 4 weeks of stress applied.

**Figure 12:**
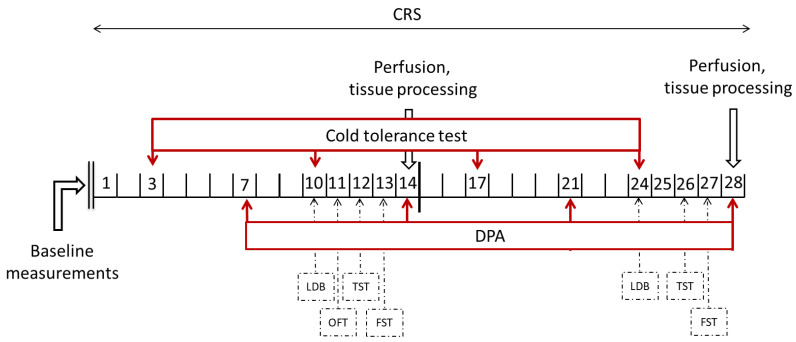
Experimental design including the timing of the baseline measurements, chronic restraint stress (CRS), nociceptive and behavioral tests and perfusion. DPA: dynamic plantar esthesiometry; LDB: light–dark box test; OFT: open field test; TST: tail suspension test; FST: forced swim test.

All nociceptive and behavioral tests were carried out in the afternoon, at least 2 h after the restraint stress. The experimenter was always blind to the stress state of the animals. Finally, the animals were anesthetized and perfused. (See the detailed protocol and time points in Figure 12.)

### 4.4. Nociceptive Measurements 

#### 4.4.1. Dynamic Plantar Aesthesiometry (DPA) 

The mechanonociceptive thresholds of the hind paws were determined with a DPA (Ugo Basile, Italy) device. The mice were placed into plastic boxes resting on a metal mesh. After a 10 min-long acclimatization period, the threshold was measured by exerting an increasing force on the plantar surface with a small-diameter metal needle (max. force: 10 g, ramp: 4 s). The force at which the animals withdrew their paws, due to pain, was recorded by the electronic unit and referred to as the mechanonociceptive threshold [97]. Three values were assessed and averaged on both paws.

#### 4.4.2. Cold Tolerance Test

The cold tolerance of the hind paws was determined by the measurement of the withdrawal latencies from icy, 0 °C water [98]. The mice were held gently, and the hind paws were separately immersed in ice-containing water at a 0 °C temperature. The time at which the animals withdrew their paws, due to pain, was recorded and referred to as the thermonociceptive threshold. The withdrawal latency time was recorded with a cut-off time of 180 s.

### 4.5. Behavioral Tests

Anxiety- and depression-like behavior was examined to exclude its confounding effect on nociceptive parameters. 

#### 4.5.1. Open Field Test (OFT)

The OFT has a wide literature in the examination of anxiety-like behavior in rodents. At the beginning of the test, the mice were placed in the center of a brightly lit open arena with a size of 48*48*55 cm. During the 5-minute-long test, the animals’ behavior was recorded with a Noldus camera system and evaluated by the EthoVision^®^ XT version 11 (Noldus, Wageningen, Netherlands) video tracking software afterwards. The time spent moving defines the locomotor activity, whereas the time spent in the border areas is proportional to anxiety [99].

#### 4.5.2. Light–Dark Box Test (LDB)

In the LDB test, the natural light aversion of mice in the lit compartment competes with the curiosity to explore the novel environment. The exploratory behavior in the light part of the box (entries to and/or time spent in the light side) is inversely proportional to the anxiety level. The box used during the test has lit (white-painted, non-covered) and dark (black-painted, roofed) compartments, with a hole between them at the floor level. The mice were placed into the light compartment at the beginning of the experiment, and their behavior was observed for 5 min, recorded with a Noldus camera system, and evaluated by the EthoVision^®^ XT version 11 (Noldus, Wageningen, The Netherlands) video tracking software afterwards. The time spent in the lit compartment, the number of entries (when all four paws of the animals were out of the dark) and the number of peeks into the light were assessed [100]. 

#### 4.5.3. Tail Suspension Test (TST)

The original TST test has been used worldwide since 1986 to evaluate depression-like behavior in rodents. We used a modified version of the original TST written down by Nielsen and his colleagues [101]. The mice were individually suspended by their tail with adhesive tape 50 cm above the surface. The ratio of escape-oriented movements (coping) and the complete lack of movement (depressive-like behavior) was determined with the immobility time, measured in the last 4 min of the 6-minute-long test period.

#### 4.5.4. Forced Swim Test (FST)

During FST, and likewise in the TST, mice react to an inescapable acute stress situation, alternating between struggling (swimming) and immobility (floating). The animals were placed individually in clear cylinders (height: 25 cm, diameter: 20 cm) containing a 19 cm depth of water (22–24 °C). The total duration of the stress exposure was 6 min, and the time of immobility (referring to the lack of escaping behavior) was measured in the final 4 min of the experiment [102].

### 4.6. Perfusion and Tissue Processing

The mice were i.p. injected with an overdose of ketamine–xylazine solution (dose: 100 and 5 mg/kg). Following deep anesthesia (checked with a toe pinch), the thoracic cavity was opened, and the animals were transcardially perfused with 0.1 M phosphate-buffered saline (PBS, pH: 7.4) and then with 4% paraformaldehyde (PFA) solution in Millonig buffer (pH: 7.4). Brains, adrenal glands and thymuses were removed, and the thymus and adrenal weight were measured. Following a one-day post-fixation in 4% PFA, six randomly selected mouse brains per group were sliced into 30 µm coronal sections with Vibratome (Leica VT1000 S, Leica Biosystems, Richmond, IL, USA). Five series of sections were made from each animal, which were placed into PBS with 0.01% aside and stored at 4 °C until further processing.

### 4.7. Immunohistochemistry: IBA1

IBA1 immunoreactivity (*n* = 6/group) was revealed by a conventional avidin–biotin immunoperoxidase protocol. After washing in PBS, free-floating brain sections were incubated sequentially in (a) 1% hydrogen peroxide (H_2_O_2_) in PBS solution for 30 min, followed by a repeated wash with PBS; (b) citrate solution at 90 °C for 10 minutes, followed by a repeated wash with PBS; (c) PBS/0.5% Triton™ X100 at room temperature for 20 min; (d) 2% normal goat serum (Vector Laboratories, Burlingame, CA) in PBS at room temperature for 30 min; (e) 2% normal goat serum in PBS with rabbit anti-IBA1 (019-19741, Wako Chemicals GmbH, Neuss, Germany, 1:10.000 dilution) primary antibody at room temperature overnight, followed by 3X wash with PBS; (f) 1.5% biotinylated goat anti-rabbit IgG (Vector Labs) at room temperature for 60 min, after repeated PBS washing; (g) avidin– biotin complex at room temperature for 60 min, followed by a PBS wash. The resulting activity was developed in 3,3-diaminobenzidine (DAB, Sigma St. Louis, MO, USA) with 0.003% H_2_O_2_ and 0.5 mg of nickel(II)–sulphate solution. The sections were mounted onto gelatine-coated slides, dehydrated in alcohols, cleared with xylene and coverslipped with DePex mounting medium.

### 4.8. Immunohistochemistry: GFAP

GFAP immunoreactivity (*n* = 5–7/group) was revealed by a conventional avidin–biotin immunoperoxidase protocol; for a detailed description, see IBA1 above. In the case of GFAP immunostaining, non-specific binding sites were blocked with 2% normal horse serum (Vector Laboratories, Burlingame, CA, USA) in PBS, and 2% normal horse serum in PBS with mouse monoclonal anti-GFAP (019-19741, Wako Chemicals GmbH, Neuss, Germany, 1:2.000 dilution) primary antibody was used. The secondary antibody was 1.5% biotinylated anti-mouse IgG (Vector Labs). In the case of GFAP immunostaining, the resulting activity was developed in 3,3-diaminobenzidine (DAB, Sigma St. Louis, MO, USA) with 0.003% H_2_O_2_ without nickel(II)–sulphate solution 

### 4.9. Immunohistochemistry Data Analysis

Quantitative analyses of integrated density, cell number and cell activation measurements were performed with ImageJ 1.48 software on micrographs taken of the areas of interest using an Olympus IX81 microscope equipped with an Olympus DP74 digital camera. 

For the histological analysis, the region of interest (ROI) was placed over each brain region (three slices/region), and integrated density (IntDen) values or IBA1+ and GFAP+ cell numbers were calculated for each picture. The IntDen values are reported as arbitrary units (AU), and the number of IBA1+ or GFAP+ cell bodies visible in the selected ROIs are shown as cell number per area. To accurately assess the degree of activation of IBA1+ microglia and GFAP+ astrocytes, a scoring system was used, as described in the literature [103]. Modified scoring was used in our study, considering both relative IBA1 and GFAP protein amounts based on morphological changes ranging from 1 (for a resting state) to 5 (fully activated state) [104].

Four pain-related brain areas were investigated: hippocampus cornu ammonis area 3 (approximately 2.18–2.46 mm caudal to bregma); primary somatosensory cortex—representation of the hind limb (approximately 0.46–0.7 mm caudal to bregma); PAG (approximately 7.3–8.3 mm caudal to bregma); CeA (approximately 1.82–2.06 mm caudal to bregma). The pictures were randomly assigned, and the investigator was also blind to the experimental groups. 

### 4.10. Statistical Analysis

The normal distribution of the data was checked by Shapiro–Wilk and Kolmogorov–Smirnov tests. In the case of datasets that did not represent a normal distribution, the Kruskal–Wallis test was applied, followed by Dunn’s test. One-way ANOVA, two-way repeated measurement (RM) or mixed model ANOVA was preferred for datasets with a normal distribution, followed by Tukey’s test. GraphPad Prism 8 software was used for statistical analysis. Data are presented as the mean ± standard error of the mean, as presented under the figures.

## Data Availability

The data that support the findings of this study are available from the corresponding author upon reasonable request.

## Abbreviations

The following abbreviations are used in this manuscript:

ANOVA	analysis of variance
CA3	hippocampus cornu ammonis area 3
CeA	central nucleus of amygdala
CRPS	complex regional pain syndrome
CRS	chronic restraint stress
DAB	diaminobenzidine
DPA	dynamic plantar esthesiometry
FST	forced swim test
GFAP	glial fibrillary acidic protein
IBA1	ionized calcium-binding adapter protein 1
ICD	International Classification of Disease
IL-1	interleukine-1
IL-1R1	interleukine-1 receptor 1
IL-6	interleukine-6
IL-8	interlekuine-8
KO	knock-out
LDB	light–dark box test
MAP	mitogen-activated protein kinase
OFT	open field test
PAG	periaqueductal gray matter
PBS	phosphate-buffered saline
PFA	paraformaldehyde
PKC	protein kinase C
S1HL	primary somatosensory cortex—representation of the hind limb
TST	tail suspension test
WHO	World Health Organization
WT	wild-type
